# Systematic review of the impact of breast‐conserving surgery on cancer outcomes of multiple ipsilateral breast cancers

**DOI:** 10.1002/bjs5.53

**Published:** 2018-05-22

**Authors:** Z. E. Winters, J. Horsnell, K. T. Elvers, A. J. Maxwell, L. J. Jones, A. M. Shaaban, P. Schmid, N. R. Williams, A. Beswick, R. Greenwood, J. C. Ingram, C. Saunders, J. S. Vaidya, L. Esserman, I. Jatoi, A. M. Brunt

**Affiliations:** ^1^ Patient‐Reported and Clinical Outcomes Research Group Surgical and Interventional Trials Unit (SITU) London UK; ^2^ SITU, Division of Surgery and Interventional Science, Faculty of Medical Sciences University College London London UK; ^3^ Centre for Tumour Biology London UK; ^4^ Centre for Experimental Cancer Medicine, Barts Cancer Institute Queen Mary University of London London UK; ^5^ Department of Breast Surgery Royal Surrey County Hospital NHS Foundation Trust Guildford UK; ^6^ Patient‐Centred and Clinical Outcomes Research Group University of Bristol, Southmead Hospital Bristol UK; ^7^ Musculoskeletal Research Unit, Translational Health Sciences, Bristol Medical School University of Bristol, Southmead Hospital Bristol UK; ^8^ Research Design Service South West University Hospitals Bristol NHS Foundation Trust Bristol UK; ^9^ Nightingale Centre Wythenshawe Hospital Manchester UK; ^10^ Department of Histopathology Queen Elizabeth Hospital Birmingham and University of Birmingham Birmingham UK; ^11^ Cancer Centre University Hospitals of North Midlands and Keele University Stoke‐on‐Trent UK; ^12^ Division of Surgery, Harry Perkins Institute of Medical Research, Fiona Stanley Hospital University of Western Australia Murdoch Western Australia Australia; ^13^ University of California San Francisco Carol Franc Buck Breast Care Centre San Francisco California USA; ^14^ Division of Surgical Oncology and Endocrine Surgery, Department of Surgery University of Texas Health Science Centre San Antonio Texas USA

## Abstract

**Background:**

The clinical effectiveness of treating ipsilateral multifocal (MF) and multicentric (MC) breast cancers using breast‐conserving surgery (BCS) compared with the standard of mastectomy is uncertain. Inconsistencies relate to definitions, incidence, staging and intertumoral heterogeneity. The primary aim of this systematic review was to compare clinical outcomes after BCS versus mastectomy for MF and MC cancers, collectively defined as multiple ipsilateral breast cancers (MIBC).

**Methods:**

Comprehensive electronic searches were undertaken to identify complete papers published in English between May 1988 and July 2015, primarily comparing clinical outcomes of BCS and mastectomy for MIBC. All study designs were included, and studies were appraised critically using the Newcastle–Ottawa Scale. The characteristics and results of identified studies were summarized.

**Results:**

Twenty‐four retrospective studies were included in the review: 17 comparative studies and seven case series. They included 3537 women with MIBC undergoing BCS; breast cancers were defined as MF in 2677 women, MC in 292, and reported as MIBC in 568. Six studies evaluated MIBC treated by BCS or mastectomy, with locoregional recurrence (LRR) rates of 2–23 per cent after BCS at median follow‐up of 59·5 (i.q.r. 56–81) months. BCS and mastectomy showed apparently equivalent rates of LRR (risk ratio 0·94, 95 per cent c.i. 0·65 to 1·36). Thirteen studies compared BCS in women with MIBC versus those with unifocal cancers, reporting LRR rates of 2–40 per cent after BCS at a median follow‐up of 64 (i.q.r. 57–73) months. One high‐quality study reported 10‐year actuarial LRR rates of 5·5 per cent for BCS in 300 women versus 6·5 per cent for mastectomy among 887 women.

**Conclusion:**

The available studies were mainly of moderate quality, historical and underpowered, with limited follow‐up and biased case selection favouring BCS rather than mastectomy for low‐risk patients. The evidence was inconclusive, weakening support for the St Gallen consensus and supporting a future randomized trial.

## Introduction

Breast cancer affects 1·7 million women annually worldwide, the majority of whom are treated surgically^1^. Clinical evidence is well established for the treatment of unifocal cancers by breast‐conserving surgery (BCS) and whole‐breast radiotherapy (RT) in preference to mastectomy^2^ ^3^. In contrast, there are no *a priori* randomized trials evaluating the clinical safety of BCS for treating multiple ipsilateral breast cancers (MIBC). MIBC are collectively defined as more than one synchronous ipsilateral cancer at diagnosis. In a national Association of Breast Surgery survey of UK breast surgeons in 2015, 91 per cent of surgeons thought that a randomized trial evaluating the safety and quality‐of‐life implications after BCS for MIBC was clinically important (Z. E. Winters, unpublished data)

Observational studies evaluating treatments for MIBC have shown wide variation in clinical outcomes. There have also been wide ranging expert opinions on optimal surgical treatments^3^ ^4^. Inherent clinical inconsistencies include variable definitions, large variation in incidences depending on the sensitivity of preoperative imaging (for example mammography *versus* MRI), underestimating the tumour load using the current TNM staging classification and unknown clinical implications of MIBC, where multifocal (MF) cancers may be clinically and genetically distinguishable from multicentric (MC) ones^5^. These issues have challenged interstudy comparisons and clinical evidence regarding treatments for MIBC.

Historically, MF cancers have been defined as more than one cancer within the same breast quadrant, whereas MC cancers are widely spaced in different quadrants. MIBC may also include ductal carcinoma *in situ* (DCIS) and invasive breast cancer^5^. However, these definitions are problematic, with no breast anatomical boundaries and variably defined distances of clinically apparently normal tissue between cancers; MF cancers are foci separated by 40 mm or les s (or no more than 20 mm), and MC cancers are foci separated by more than 40 mm (or over 20 mm) or tumours in different quadrants^6^, ^7^, ^8^. According to the College of American Pathologists guidelines^9^, characterization of only the largest lesion in MIBC is sufficient, provided that all cancers have the same tumour grade. Despite this, several authors^10^ ^11^ have suggested revising the current TNM staging system^12^, potentially avoiding underestimation of the overall disease burden for MIBC. Recently, Desmedt and colleagues^8^ suggested that genomically heterogeneous cancers tended to be situated further apart (MC cancers), whereas those closer together (MF cancers) showed intertumoral homogeneity. Molecular characterization of each cancer focus to distinguish between MC and MF cancers may be important in future classifications^8^. Intertumoral heterogeneity in MIBC has been reported in 11–27 per cent of patients^8^ ^13^, ^14^. Generally, evaluation of the largest cancer using standard immunohistochemical (IHC) biomarkers (oestrogen and progesterone receptors, human epidermal growth factor receptor 2 (HER2) and Ki‐67) is performed^3^ ^9^, ^15^. However in future, the potential to use extended IHC biomarkers and whole‐genome sequencing may increase the recognition of intertumoral heterogeneity^14^ ^16^, ^17^, ^18^, with prognostic implications^19^, ^20^, ^21^, ^22^.

Clinically occult cancers may remain dormant, or may be treated adequately by adjuvant whole‐breast RT after BCS^8^. The incidence of clinically and radiologically detected MIBC ranges from 10 to 24 per cent of all breast cancers^20^, ^21^, ^22^, ^23^, increasing with time from earlier to later studies. This apparent doubling in incidence of MIBC over the 10 years between 1990 and 2000 may be due in part to improved breast imaging (digital mammography, ultrasonography and MRI) and increased screening^24^. Standard imaging of MIBC may include digital mammography, ultrasound examination and MRI, with biopsy confirmation of any additional suspected cancers on MRI to minimize their misdiagnosis, which occurs in 30 per cent of ‘lesions’^24^ ^25^. Studies^6^ ^10^, ^11^ ^19^, ^20^, ^21^ ^23^, ^26^ are conflicting on the prognostic implications of MIBC, with suggestions of increased axillary lymph node involvement latterly using sentinel lymph node biopsy (SLNB), and with worse overall outcomes than those for unifocal cancers^19^ ^22^, ^23^. Increased rates of locoregional recurrence (LRR)^5^ and breast cancer events^22^ ^23^, ^27^ ^28^ secondary to BCS for MIBC have been reported, but vary widely across studies. Recently, a clear majority of the St Gallen expert consensus panel^3^ expressed the opinion that it was possible to treat MF and MC cancers with BCS, where there was margin clearance and whole‐breast RT was planned. However, this opinion was not contextualized to clinical outcomes specifically differentiating MF and MC cancers, or comparable outcomes following mastectomy as the standard of care. Current guidelines are concordant regarding adjuvant treatments (chemotherapy, endocrine therapy, targeted therapy and RT) informed by tumour subtypes^3^ ^5^.

This systematic review critically appraises the levels of clinical evidence, and whether these support or weaken the case on which expert opinion is based in support of BCS. The primary aim was to compare disease‐specific outcomes following BCS *versus* mastectomy for treating MIBC (including MF and MC cancers). Studies comparing BCS for treating MIBC and unifocal disease were also evaluated.

## Methods

A protocol was developed that followed the PRISMA statement for review methods and reporting^29^. A PICOS (participants, interventions, comparators, outcomes and study designs) sequence was used to describe the included studies. In this systematic review, the term MIBC refers to both MF and MC cancers. Where individual studies referred specifically to MF or MC cancers, these definitions were retained.

### Literature search strategy

Web‐based search engines MEDLINE, Embase, PsycINFO, ISI Web of Knowledge and Cochrane databases were interrogated using keywords (*Appendix* S1, supporting information). The search was limited to human studies published in English from May 1988 to July 2015. Abstracts and conference reports were excluded owing to difficulties in evaluating incomplete information. Duplicate records were excluded. Two independent reviewers screened titles and abstracts for eligibility using predetermined criteria. Reference lists of screened articles and reviews were searched manually to identify further relevant studies. The intention was to focus on RCTs and non‐randomized longitudinal cohort studies, and this was extended to include all study designs.

### Data extraction

A standardized data pro forma was created including study design, nature of data accrual, number of centres, years of data collection, study inclusion and exclusion criteria, diagnostic methods, clinical outcomes (LRR, disease‐free survival (DFS) and overall survival), duration of follow‐up, pathological details, adjuvant treatment and non‐clinical outcomes (*Appendix* S2, supporting information). All data were evaluated independently by two authors, and discrepancies resolved by the senior author.

### Inclusion and exclusion criteria and outcomes

Included studies were those evaluating women aged at least 18 years with invasive cancer and/or DCIS diagnosed as MIBC before operation or after surgery (histological diagnosis). Eligible interventions comprised BCS, either primary or secondary to neoadjuvant treatment. To be included, studies required a direct comparison of BCS with mastectomy for MIBC (primary aim), or a comparison of BCS for MIBC *versus* BCS for unifocal cancers (secondary aim). There were no exclusion criteria relating to minimum numbers of participants or duration of follow‐up.

Other inclusion criteria required the study to be primary research as opposed to audit, and to include a minimum of one relevant clinical outcome such as: LRR (local relapse‐free, clinical local recurrence), distant metastasis (distant disease), DFS (disease‐free survival) or overall survival (breast cancer‐specific disease, overall metastases, breast cancer‐specific survival).

### Study quality

Risk of study bias was assessed using the Newcastle–Ottawa scale (NOS)^30^ ^31^. The NOS comprises a semiquantitative assessment of study quality, using eight separate measures. These measures score the study based on selection of patients, comparability of groups and reporting of outcomes. The score ranges from 0 to 9. Studies scoring 7 or more are considered high quality, those scoring 4–6 are of moderate quality, and those with a score of less than 4 are of poor quality. Two reviewers scored each study independently, with discussion and re‐evaluation of scoring discrepancies resulting in consensus.

### Data analysis

Data on recurrence rates were analysed using a fixed‐effects model. Risk ratios and 95 per cent confidence intervals were calculated with Revman 5.3 software (Nordic Cochrane Centre, Copenhagen, Denmark) and presented as a forest plot. Heterogeneity among studies was assessed by means of the *I* 2 statistic.

## Results

### Study selection

**Figure 1:**
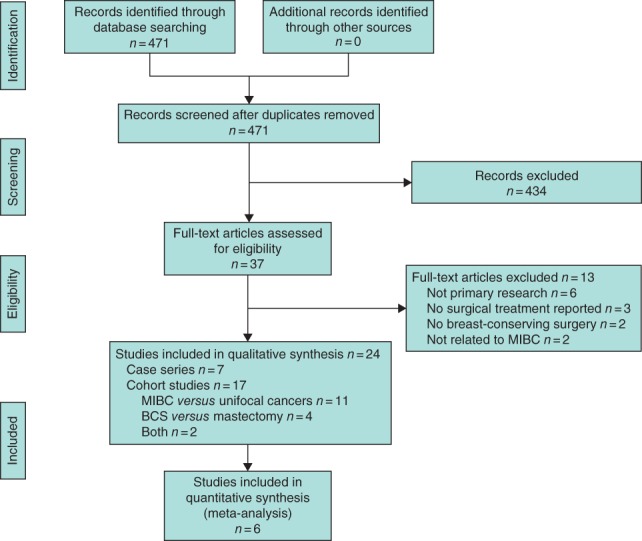
Selection of articles for review. MIBC, multiple ipsilateral breast cancers; BCS, breast‐conserving surgery

Titles and abstracts of 471 citations were identified electronically (*Fig*. 1) After review of 37 full‐text articles, 24 primary papers were retained. Of the 13 articles excluded, six did not report primary research (review articles), three did not report on surgical interventions, two did not report on BCS for MIBC and two were not focused on MIBC.

### Study design

There were no RCTs and there was a paucity of published data addressing the primary aim. The 24 studies had retrospective observational designs, comprising 17 comparative studies^20^, ^21^, ^22^, ^23^ ^27^, ^32^, ^33^, ^34^, ^35^, ^36^, ^37^, ^38^, ^39^, ^40^, ^41^, ^42^, ^43^ and seven case series^28^ ^44^, ^45^, ^46^, ^47^, ^48^, ^49^ (Table 1; Table S1, supporting information). The studies included a total of 3537 women with MIBC undergoing BCS: 2677 with tumours defined as MF, 292 as MC, and 568 not defined as either MF or MC, but reported as MIBC. Assessment of the observational studies using the NOS showed that three^20^ ^27^, ^43^ were of high quality (score at least 7) and 14^21^, ^22^, ^23^ ^32^, ^33^, ^34^, ^35^, ^36^, ^37^, ^38^, ^39^, ^40^, ^41^, ^42^ of moderate quality (score 4–6) (Table S2, supporting information).

**Table 1:** Summary of study characteristics, treatments and clinical outcomes of primary studies comparing breast‐conserving surgery with mastectomy

Reference Study interval and location NOS score	Cancers included FU (months)*	Group differences in CP	No of patients (MF; MC) Other treatments Pathology		Outcomes	
			BCS	Mastectomy	BCS‡	Mastectomy
Nos et al. 32 1983–1989 France 1 centre NOS 4	n.a. FU 101 (86–129)	Significant differences in age and T category	56 (56; 0) Radiotherapy, chemotherapy, endocrine IDC 79%, ILC 13%	132 Radiotherapy, chemotherapy, endocrine IDC 83%, ILC 8%	5‐year LRR: 11% 10‐year LRR: 23% 5‐year DM: 18% 10‐year DM: 28% 5‐year OS: 94% 10‐year OS: 73%	5‐year LRR: 11% 10‐year LRR: 14% 5‐year DM: 18% 10‐year DM: 35% 5‐year OS: 89% 10‐year OS: 65%
Kaplan et al. 33, † 1989–2002 USA 2 centres NOS 4	MIBC, diagnosed before surgery FU 45 (1–143)	No significant differences	36 Radiotherapy, chemotherapy, endocrine IDC 72%, ILC 19%, DCIS 8%	19 Radiotherapy, chemotherapy, endocrine IDC 68%, ILC 21%, DCIS 11%	5‐year LRR: 1 of 36 (3%) (P = 0.54) DM: 1 of 36 (3%) (P = 0·20) OS: 36 of 36 (100%)	5‐year LRR: 0 of 19 (0%) DM: 1 of 19 (5%) OS: 19 of 19 (100%)
Lim et al. 34 1990–2003 South Korea 1 centre NOS 6	MF FU 59 (1–177) Mastectomy: 65 (6–196)	HER2+ (P = 0·007) T2 (P = 0·006)	147 (147; 0) Radiotherapy, endocrine IDC 97%, ILC 3%	331 Radiotherapy, endocrine IDC 96%, ILC 4%	5‐year LRR: 3 of 147 (2.0%) (P = 0·38) 5‐year DFS: 89% (P = 0·45) 5‐year OS: 93·4% (P = 0·21)	5‐year LRR: 3 of 331 (0·9%) 5‐year DFS: 92% 5‐year OS: 94·5%
Kadioğlu et al. 35 2002–2011 Turkey 1 centre NOS 5	MF, diagnosed by histology FU 55 (10–102)	No. of foci (P = 0·001) LVI (P = 0·04) LN positivity (P = 0·002) TNM stage (P = 0·01) HER2+ (P = 0·03)	119 (119; 0) Radiotherapy, chemotherapy, endocrine IDC 71%, ILC 12%	103 Radiotherapy, chemotherapy, endocrine IDC 69%, ILC 8%	5‐year LRR: 6 of 119 (5·0%) (P = 0·06) 5‐year OS: 92%, median 95 (range 91–99) months (P < 0·001)	5‐year LRR: 6 of 103 (5·8%) 5‐year OS: 72%, median 73 (range 68–78) months
Neri et al. 20, † Italy 1991–2005 1 centre NOS 7	MF diagnosed before surgery FU 88 (11–248)	n.a.	36 (36; 0) Radiotherapy, chemotherapy endocrine	155 Radiotherapy, chemotherapy, endocrine	7‐year LR: 3 of 36 (8%) 7‐year LLR: 5 of 36 (14%) 7‐year RR: 2 of 36 (6%) DM: 7 of 36 (19%)	7‐year LR: 12 of 155 (7·7%) 7‐year LLR: 23 of 155 (14·8%) 7‐year RR: 11 of 155 (7·1%) DM: 42 of 155 (27·1%)
Yerushalmi et al. 43, † 1989–2005 South Korea 5 regional centres NOS 7	MIBC, diagnosed before surgery FU 93	T, N category (P < 0·001) EIC (P < 0·001) Positive margins (P < 0·001)	300 Radiotherapy, chemotherapy, endocrine IDC 88%, ILC 12%	887 Radiotherapy, chemotherapy, endocrine IDC 92%, ILC 6%	17 of 300 (5·7%)	58 of 887 (6·5%)

* Values are mean (range). All studies were retrospective;

† prospective database.

‡ P values are for comparison of breast‐conserving surgery (BCS) versus mastectomy. NOS, Newcastle–Ottawa Scale; FU, follow‐up; CP, clinical pathology; MF, multifocal; MC, multicentric; n.a., not available; IDC, invasive ductal cancer; ILC, invasive lobular cancer; LRR, locoregional recurrence; DM, distant metastasis; OS, overall survival; MIBC, multiple ipsilateral breast cancers; DCIS, preinvasive ductal cancer in situ; HER2, human epidermal growth factor receptor 2; T2, tumour size 20–50 mm; DFS, disease‐free survival; LVI, lymphovascular invasion; LN, lymph node; LR, local recurrence; RR, regional recurrence; EIC, extensive preinvasive cancer or DCIS. Further details of the studies can be found in Tables S1–S3 (supporting information).

### Participants

Seventeen studies^20^ ^21^, ^27^ ^28^, ^32^ ^34^, ^35^, ^36^, ^37^ ^40^, ^41^, ^42^ ^44^, ^46^, ^47^, ^48^, ^49^ were from single centres, four^33^ ^38^, ^39^ ^45^ involved two centres, and there were three multicentre studies^22^ ^23^, ^43^ comprising a regional cancer registry (British Columbia Cancer Agency)^43^, a German regional study group (BRENDA Study Group: Breast Cancer Care under Evidence‐based Guidelines)^23^ and a substudy within three multicentre RCTs of neoadjuvant chemotherapy (German Breast Group (Gepar) trials)^22^ (Table 1; Table S1, supporting information).

Studies included patients recruited between 1968 and 2010, with only two^22^ ^35^ including patients treated after 2000. The median group size of patients with MIBC receiving BCS was 61 (i.q.r. 35–169) in comparative studies and 22 (14–43) in case series. Diagnostic methods for evaluating MIBC were recorded in six of the seven case series^44^, ^45^, ^46^, ^47^, ^48^, ^49^ and nine^27^ ^32^, ^33^ ^35^, ^36^, ^37^, ^38^, ^39^, ^40^ of the 17 comparative studies. In these 15 studies, 258 of 592 patients (43·6 per cent) were diagnosed by postoperative pathology and 294 (49·7 per cent) before surgery: clinical examination (129, 21·8 per cent), radiology (103, 17·4 per cent) or undefined methods (62, 10·5 per cent). The remaining 40 patients (6·8 per cent) were classified as having MIBC based on lesion detection at surgery^32^ ^38^, ^39^. Where preoperative diagnostic methods were defined, ultrasound imaging was reported in four^27^ ^33^, ^40^ ^46^ and MRI in three^35^ ^47^, ^49^ studies.

Data were collected retrospectively in the case series, whereas six comparative studies^20^, ^21^, ^22^ ^27^, ^33^ ^43^ used a prospective database to identify patients with MIBC treated by BCS. In the 11 other comparative studies, data were derived from postoperative histology reports based on pathologically determined as opposed to clinically detected disease. Ataseven and co‐workers^22^ omitted prerequisite pathological confirmation of each cancer. Studies reported varied inclusion criteria for MIBC. Twelve studies^20^ ^23^, ^27^ ^32^, ^34^ ^36^, ^37^ ^40^, ^42^ ^43^, ^48^ ^49^ based inclusion on TNM staging, with three of these^23^ ^32^, ^42^ mandating inclusion by size of the largest cancer. Another six studies^21^ ^28^, ^39^ ^44^, ^46^ ^47^ used the feasibility of BCS to define eligibility. The remaining studies included: macroscopically separate tumours^33^ ^35^, ^45^, synchronous ipsilateral breast cancers on gross pathological review over and above microscopy^38^, patients with DCIS undergoing BCS^41^ and recruitment within the Gepar trials^22^.

### Clinical and pathological characteristics

Significant differences between groups in clinical and pathological characteristics (age, tumour type, DCIS, TNM stage, receptor status (oestrogen receptor, progesterone receptor, HER2), lymphovascular invasion, margins) were reported in four studies^32^ ^34^, ^35^ ^43^ of BCS versus mastectomy for MIBC, and in four studies^27^ ^37^, ^39^ ^43^ of BCS for MIBC versus unifocal cancers (Table S3, supporting information). Intergroup comparisons showed that prognostic factors were worse among patients who underwent mastectomy than those who underwent BCS, and among those with MIBC compared with those who had unifocal disease. Yerushalmi and colleagues^43^ used a matched analysis (3 : 1) of clinicopathological characteristics, accounting for three times more unifocal cancers than MIBC. Retrospective study designs made it difficult to compare clinical data, compounded by significantly larger numbers of unifocal cancers^20^, ^21^, ^22^, ^23^ ^41^, ^42^. With the exception of one recent study^22^, no studies reported the distribution of molecular subtypes (luminal A, luminal B, non‐luminal HER2, basal and triple‐negative). Overall, five studies^23^ ^27^, ^34^ ^35^, ^43^ used statistical regression methodologies adjusting for baseline co‐variables.

### Definitions of MIBC

Definitions of MF or MC tumours were described in 21^20^, ^21^, ^22^, ^23^ ^27^, ^28^ ^32^, ^34^ ^35^, ^37^ ^39^, ^40^, ^41^, ^42^, ^43^, ^44^, ^45^, ^46^, ^47^, ^48^, ^49^ of 24 studies. Eleven studies assessed MIBC as a single group^33^ ^36^, ^37^, ^38^, ^39^, ^40^ ^42^, ^43^, ^44^, ^45^ ^47^, and 13^20^, ^21^, ^22^, ^23^ ^27^, ^28^ ^32^, ^34^ ^35^, ^41^ ^46^, ^48^ ^49^ evaluated BCS in MF and MC cancers separately (Table 1; Table S3, supporting information).

### Clinical outcomes

Clinical outcomes are summarized in Table 1 and detailed in Table S4 (supporting information). Eleven^21^ ^22^, ^27^ ^36^, ^37^, ^38^, ^39^, ^40^, ^41^, ^42^, ^43^ of 17 comparative studies evaluated clinical outcomes of BCS for both MIBC and unifocal cancers; only four studies^23^ ^32^, ^33^ ^35^ exclusively compared BCS with mastectomy for MIBC. Lim and colleagues^34^ and Neri et al. 20 evaluated outcomes of both MIBC and unifocal cancers independent of the type of surgery. Of the 24 studies, five^20^ ^23^, ^27^ ^32^, ^43^ reported actuarial 10‐year clinical outcomes, despite 10 years being the optimal period of follow‐up according to the Association of Breast Surgery guidelines^50^. Overall, the studies reported a median follow‐up of 60 (i.q.r. 53–73) months.

### Interventions

Various descriptions of the type of BCS were used (Table 1; Table S1, supporting information). Fourteen studies^21^, ^22^, ^23^ ^28^, ^32^ ^34^, ^35^ ^39^, ^40^, ^41^, ^42^, ^43^, ^44^ ^47^ referred to BCS and three^36^, ^37^, ^38^ to wide local excision. Others referred to partial mastectomy^20^ ^45^, quadrantectomy^46^ ^48^, segmentectomy^27^ or lumpectomy^33^ ^49^. The extent of acceptable microscopic margin status following BCS was defined in 18^21^, ^22^, ^23^ ^27^, ^28^ ^33^, ^34^, ^35^, ^36^, ^37^, ^38^, ^39^, ^40^, ^41^ ^44^, ^45^, ^46^ ^48^ actuarial studies. Differing definitions of microscopically clear radial cancer margins were used. Commonly, this comprised radial margins of at least 1 mm^23^ ^27^, ^28^ ^33^, ^34^ ^40^, ^44^ ^48^, although two studies^5^ ^46^ used 2 mm or more and one^39^ required margins of at least 5 mm. Other studies referred to ‘grossly excised’^36^, ^37^, ^38^, ‘tumour‐free margins’^22^ and ‘clear at the inked margin’^41^. In one study^21^, margins were defined as ‘close’ if less than 2 mm from the ‘cut edges’, and another^35^ based margin re‐excisions on clinicians' assessments.

### Adjuvant treatments

#### *Adjuvant radiotherapy*

All 24 studies reported using postoperative RT after BCS. Completion of RT after BCS was described in 15 studies^20^ ^21^, ^23^ ^27^, ^32^ ^35^, ^36^ ^38^, ^39^, ^40^, ^41^ ^43^, ^44^, ^45^, ^46^. In three studies^22^ ^37^, ^42^ it was not possible to define the extent of RT or dose fractionation used. The RT regimen was described in 11 studies^23^ ^32^, ^36^, ^37^, ^38^, ^39^, ^40^ ^44^, ^45^, ^46^, ^47^, with a tumour bed boost RT reported in eight^23^ ^32^, ^36^ ^37^, ^39^ ^40^, ^45^ ^46^ of these. There was no mention of more than one lumpectomy bed receiving a tumour bed RT boost in MC cancers; however, there were only 223 MC cancers treated by BCS (Table 1; Table S1, supporting information).

#### *Adjuvant endocrine therapy*

Twenty studies^20^, ^21^, ^22^, ^23^ ^27^, ^28^ ^32^, ^33^, ^34^, ^35^ ^39^, ^41^, ^42^, ^43^, ^44^, ^45^, ^46^, ^47^, ^48^, ^49^ reported using adjuvant endocrine treatments. Eighteen^20^ ^21^, ^27^ ^28^, ^32^, ^33^, ^34^, ^35^ ^39^, ^41^, ^42^, ^43^, ^44^, ^45^, ^46^, ^47^, ^48^, ^49^ reported patient compliance for endocrine therapy, with a median of 68 (i.q.r. 43–87) per cent. Four studies^34^ ^47^, ^48^, ^49^ reported endocrine treatment of oestrogen receptor‐positive cancers, with a median compliance rate of 92 (78–96) per cent.

#### *Adjuvant chemotherapy*

Twenty studies^20^ ^22^, ^23^ ^27^, ^28^ ^32^, ^33^ ^35^, ^37^, ^38^, ^39^, ^40^ ^42^, ^43^, ^44^, ^45^, ^46^, ^47^, ^48^, ^49^ described the use of chemotherapy, with 18 reporting percentages of patients who received it; this varied widely. Three studies^22^ ^39^, ^40^ reported that all patients received some form of chemotherapy. The overall median percentage of patients receiving chemotherapy was 57 (i.q.r. 43–77) per cent^20^ ^22^, ^27^ ^28^, ^32^ ^33^, ^35^ ^38^, ^39^, ^40^ ^42^, ^43^, ^44^, ^45^, ^46^, ^47^, ^48^, ^49^. Eight studies^22^ ^35^, ^38^, ^39^, ^40^ ^44^, ^45^ ^47^ described chemotherapy schedules and proportions of women treated, and 11 others^20^ ^21^, ^27^ ^28^, ^32^ ^33^, ^42^ ^43^, ^46^ ^48^, ^49^ reported proportions of patients alone, without chemotherapy regimens. Only two studies^22^ ^40^ described neoadjuvant chemotherapy.

### Clinical cancer outcomes in cohort studies

Reported clinical outcomes varied widely regarding clinical endpoints and duration of follow‐up (Table 1; Table S4, supporting information). Clinical outcomes reported were: LRR^20^, ^21^, ^22^ ^27^, ^32^, ^33^, ^34^, ^35^, ^36^, ^37^, ^38^, ^39^, ^40^, ^41^, ^42^, ^43^, overall survival^20^ ^27^, ^32^, ^33^, ^34^, ^35^ ^38^, ^40^ ^43^, occurrence of distant metastases^20^ ^32^, ^33^ ^36^ and DFS^22^ ^23^, ^27^ ^34^. Eleven studies^21^ ^32^, ^33^, ^34^, ^35^, ^36^, ^37^, ^38^, ^39^, ^40^ ^42^ reported outcomes at 5 years or just over; three^32^ ^41^, ^43^ reported outcomes at 5 and 10 years; one^23^ reported at 10 years only; and the other three studies reported outcomes at 3 years^22^, 7 years^20^ and 9 years^27^. The median duration of follow‐up in all studies was 60 (i.q.r. 53–73) months.

### Clinical cancer outcomes in case series

Six^28^ ^44^, ^45^, ^46^, ^47^ ^49^ of seven case series evaluated clinical outcomes after BCS for MIBC with rates of ‘local recurrences’ (LRR, local relapse‐free and cumulative local events) ranging from 0 to 5·1 per cent over a median follow‐up of 53 (i.q.r. 34–72) months (Table S4, supporting information). The 5‐year LRR data were weaker, with Gentilini and colleagues^28^ reporting a 6‐year actuarial LRR rate of 5·1 per cent (24 of 476) after BCS, without a comparator group. Distant metastasis rates ranged from 4·5 to 11 per cent during follow‐up. Median overall survival rates in four studies^28^ ^44^, ^48^ ^49^ ranged from 89 to 100 per cent.

### Clinical cancer outcomes after breast‐conserving surgery versus mastectomy for MIBC

Six^20^ ^32^, ^33^, ^34^, ^35^ ^43^ of seven studies reported clinical outcomes for BCS versus mastectomy for MIBC, which was the primary aim of the review, with a median follow‐up of 59·5 (i.q.r. 56–81) months (Table 1; Table S4, supporting information). The largest of the seven studies was part of the multicentre BRENDA cohort study^23^, but did not provide raw data for comparison. This was scored as having moderate quality based on analyses of clinical subgroups, judged to be adherent to German guidelines or not. Adherence to guidelines meant that BCS was contraindicated for MC cancers^23^. Non‐conformance with guidelines resulted in 12·9 per cent of MC cancers (60 of 464) being treated with BCS, compared with 46·8 per cent (217 of 464) undergoing mastectomy^23^. LRR was reported in five studies^20^ ^32^, ^33^, ^34^, ^35^, distant metastases in three^20^ ^32^, ^33^, overall survival in four^32^, ^33^, ^34^, ^35^ and DFS in two^23^ ^34^.

#### *Local recurrence*

**Figure 2:**
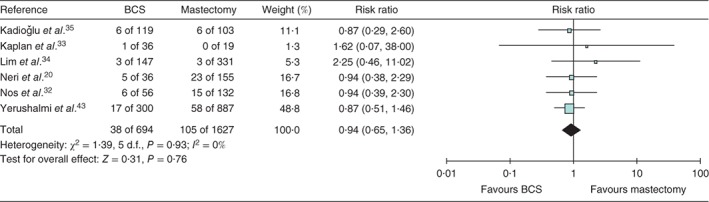
Risk ratio for locoregional recurrence after breast‐conserving surgery (BCS) versus mastectomy. An inverse‐variance fixed‐effect model was used for meta‐analysis. Risk ratios are shown with 95 per cent confidence intervals. Reference 23 was not included in this analysis as no raw data were available

Six studies^20^ ^32^, ^33^, ^34^, ^35^ ^43^ reported LRR rates ranging from 2 to 23 per cent after BCS, with apparently similar rates of LRR for BCS compared with mastectomy (risk ratio 0·94, 95 per cent c.i. 0·65 to 1·36) (Fig. 2). There was no heterogeneity in these studies, which may reflect similar case selection biases with surgeons choosing BCS for low‐risk patients and mastectomy for high‐risk cases. Overall, the results should be viewed with caution because they may be compromised by study quality.

The historical study of Yerushalmi and colleagues^43^ reported the potential clinical equivalence of mastectomy in 887 patients compared with standard BCS in 300 patients, with 10‐year LRR rates of 6·5 per cent (58 of 887) *versus* 5·7 per cent (17 of 300) respectively (*P* = 0·95). Five‐year LRR rates of MIBC in this study were 4·5 per cent after mastectomy *versus* 2·5 per cent after BCS^43^.

#### *Survival*

Wolters and colleagues^23^ concluded that treatment of MF cancers according to German guidelines by BCS (683 of 1398, 48·9 per cent) *versus* mastectomy (329 of 1398, 23·5 per cent) showed no significant differences in 5‐year recurrence‐free survival. Neri and co‐workers^20^ showed that MF cancers were a significant independent predictor of worse breast cancer‐specific survival for BCS (hazard ratio (HR) 3·88, 95 per cent c.i. 1·06 to 14·12; *P* = 0·026) and mastectomy (HR 2·72, 1·15 to 6·48; *P* = 0·023). Kadioğlu *et al*.^35^ reported significantly better 5‐year survival of 92 per cent (median 95 (range 91–99) months) after BCS in 119 patients, compared with 72 per cent (median 73 (68–78) months) after mastectomy in 103 patients (*P* < 0·001). Multivariable analyses in the latter study, accounting for intergroup differences, subsequently showed no significant effects on outcomes between types of surgery (*P* = 0·07)^35^. Similarly, Nos and colleagues^32^, Kaplan and co‐workers^33^ and Lim *et al*.^34^ reported no differences in overall survival, DFS or distant metastases by type of surgery.

### Clinical outcomes after breast‐conserving surgery for MIBC versus unifocal cancers

*Table* S4 (supporting information) provides data on outcomes after BCS for MIBC *versus* unifocal cancers.

#### *Local recurrence*

Twelve of 13 studies reported LRR rates after BCS for MIBC ranging from 2 to 40 per cent at a median follow‐up of 64 (i.q.r. 57–73) months. Two historical studies^36^ ^37^ showed significantly higher LRR rates after BCS for MIBC compared with those for unifocal cancers. More recently, Chung *et al*.^27^ reported significantly worse 9‐year LRR rates of 6·1 per cent (10 of 164) in patients with MIBC compared with 0·6 per cent (6 of 999) in patients with unifocal disease (*P* = 0·001). Using a matched‐pair analysis, Yerushalmi and colleagues^43^ reported equivalent actuarial 10‐year local recurrence rates of 5·6 per cent for MIBC (MF and MC) among 300 patients treated by BCS *versus* 4·3 per cent for unifocal cancer among 11 683 patients after BCS (HR 1·09, 95 per cent c.i. 0·55 to 2·16; *P* = 0·78). Furthermore, no differences were shown for 10‐year LRR between the groups (*Table* S4, supporting information). Neri *et al*.^20^ reported significantly worse 7‐year LRR for MIBC treated by BCS (2 of 36, 6 per cent) compared with BCS for unifocal cancers (8 of 491, 1·6 per cent) (*P* = 0·050). This study reported 304 disease recurrences at a median follow‐up of 7·3 years (range 11–248 months), with a median time to relapse of 32 months, and 172 deaths from breast cancer^20^.

### Survival

Nine‐year DFS was reported in two studies^27^ ^34^ (*Table* S4, supporting information). Chung and co‐workers^27^ reported a significantly worse 9‐year DFS rate of 89·3 per cent (14 breast cancer‐related events among 164 patients) for MIBC treated by BCS *versus* 97·7 per cent (17 of 999) following BCS for unifocal cancers, with a HR of 5·86 (95 per cent c.i. 2·57 to 13·3; *P* < 0·001) in multivariable analysis. A substudy^22^ within the GeparTrio randomized trials evaluated the impact of neoadjuvant chemotherapy on clinical outcomes of patients with unifocal, MF and MC cancers. The choice of either BCS or mastectomy was based on clinical cancer responses and the surgeon's discretion. This substudy demonstrated that 5‐year local relapse‐free survival (LRFS) rates in all patients were significantly adversely affected by focality; MC cancers alone had the worst LRFS rates of 90·4 per cent, compared with 95·1 per cent for MF and 92·9 per cent for unifocal cancers. Lower LRFS rates persisted for MC cancers after mastectomy (*P* = 0·030), although this group presented with significantly worse disease in terms of TNM stage and HER2 positivity (*P* < 0·001)^22^. Survival outcomes (LRFS) were not inferior for MC (*P* = 0·844) and MF (*P* = 0·430) cancers compared with unifocal cancers after complete pathological responses to neoadjuvant chemotherapy^22^. Neri and colleagues^20^ identified MIBC as a significant independent prognostic factor for breast cancer‐specific survival (HR 1·64, 95 per cent c.i. 1·05 to 2·57; *P* = 0·029) using multivariable analyses. Overall actuarial breast cancer‐specific survival rates (independent of type of surgery) for MIBC were 89·7 per cent at 5 years and 79·8 per cent at 10 years.

Distant metastases were reported in two articles^20^ ^36^. Leopold *et al*.^36^ reported a 5‐year rate of distant metastasis of 40 per cent (4 of 10) for MIBC treated by BCS. In both papers, distant disease was more common in the MIBC group. The results reported by Neri and co‐workers^20^ were statistically significant (19 *versus* 11·2 per cent for MIBC *versus* unifocal cancers respectively; *P* = 0·030).

Overall survival was reported in six studies^20^ ^27^, ^34^ ^38^, ^40^ ^43^. Two studies demonstrated significantly worse survival for MIBC compared with unifocal cancers at a mean follow‐up of 88 months (HR 3·88, 95 per cent c.i. 1·06 to 14·12; *P* = 0·020)^20^ and 112 months (85·8 *versus* 98·4 per cent; *P* < 0·001)^27^. Of the remaining four studies, two^34^ ^38^ reported worse outcomes, and two^40^ ^43^ better outcomes for MIBC, with no significant differences. Although not statistically significant, a further six studies^21^ ^38^, ^39^ ^41^, ^42^, ^43^ reported trends towards worse outcomes after BCS for MIBC compared with unifocal cancers.

## Discussion

This systematic review attempted to appraise the published literature critically regarding the impact of BCS in treating MIBC, compared with the standard of mastectomy with or without breast reconstruction. Overall, there was limited evidence of moderate quality supporting the clinical equivalence of BCS *versus* mastectomy for treating MIBC. Factors limiting the quality of evidence were study designs, heterogeneous clinical outcomes, and few if any representative studies of use of BCS to treat MC tumours compared with MF cancers. The inclusion of exclusively pathological diagnosis in some studies and the complexity of surgical case selection were major limiting factors inherent in the study designs. Other factors were non‐comparability of statistical methodologies, low patient numbers by type of surgery (particularly for MC cancers) and limited duration of follow‐up. In the context of current treatments, most studies were historical, with poor reporting of adjuvant chemotherapy^22^. Two studies^22^ ^40^ evaluated neoadjuvant chemotherapy and BCS in MIBC. Most did not address the primary aim of this review, but compared BCS for MIBC *versus* unifocal cancers. The apparent lack of significant intergroup differences in the rates of LRR may allow clinical equipoise and support the rationale for a randomized trial. A National Institute for Health Research (NIHR)‐funded randomized trial would evaluate the non‐inferiority in terms of LRR after BCS for MF and/or MC cancers (MIBC) compared with mastectomy.

Most studies included in this review were at high risk of bias^30^ ^31^, ^51^ ^52^. Although one‐quarter of studies used a prospective database, none of these published their protocols or defined their core clinical outcome sets^52^ ^53^, or reported study size calculations for the surgical groups^54^. Other markers of study quality were lacking, such as ethical approvals and conflicts of interest^30^ ^31^, ^51^.

Older studies did not report therapeutic mammaplasty (TM) techniques^55^, ^56^, ^57^. TM as a form of BCS for treating unifocal cancers has become more common over the past 5 years. However, evidence is lacking for its use in the treatment of MIBC^55^, ^56^, ^57^. TM techniques comprise either extended breast tissue excisions for cancer(s) with simple reapproximation of breast tissue (level 1 reconing) or a therapeutic reduction mammaplasty (level 2)^56^ ^57^. Currently, TM is the standard best practice for optimizing cosmetic outcomes after extended breast tissue excisions relative to breast volume. Recently, a small case series^58^ (68 patients) describing BCS for 20 patients with MF cancers was reported. In principle, treating MC cancers using two or more separate wide local excisions combined with TM merits future investigation, particularly in the context of RT boost(s) to one or more tumour beds. A meta‐analysis^56^ of a non‐randomized comparison between 3165 TM procedures with standard BCS in 5494 patients with unifocal cancers showed that the former significantly reduced rates of cancer margin positivity (*P* < 0·001) and surgical re‐excisions (*P* < 0·001). Recently, the St Gallen panel^2^ recommended a minimal acceptable surgical margin of ‘no ink on invasive tumour or DCIS’. Other interventions significantly reducing intraoperative tumour margin positivity have been described: digital specimen radiology (*P* = 0·012 for digital *versus* conventional mammography)^59^, tumour margin cavity shaves^60^ and real‐time cancer margin assessments^61^ ^62^. Ataseven and colleagues^22^ reported that neoadjuvant chemotherapy‐induced pathological complete cancer response rates increased the surgical options for BCS without compromising clinical outcomes, an approach requiring future investigation. Future TM approaches for treating MIBC should recommend standardized operating procedures, involving tumour bed clips to facilitate image‐guided RT^63^, ^64^, ^65^, ^66^, ^67^, ^68^, ^69^. A minority of studies (8 of 24) in the present review referred to tumour bed boost RT following BCS for MIBC. The feasibility of administering one or more tumour bed RT boosts after TM in MC cancers will be evaluated in future^63^ ^69^.

MIBC may more frequently be associated with poor prognostic factors compared with unifocal disease^5^ ^20^, ^22^ ^23^, ^26^. Coombs and Boyages^10^ recommended using aggregate cancer dimensions, thereby upstaging most MIBC to more advanced stages, with rates of lymph node positivity stage‐for‐stage comparable to those of unifocal cancers. Positive lymph node involvement was reported in 44–50 per cent of MIBC cases, compared with 38 per cent of unifocal cancers^5^ ^10^, ^20^ ^23^, ^67^ ^70^. Dual‐localization SLNB is accurate diagnostically in MIBC^70^. A subset of women with MIBC (342, 8·5 per cent) in the European Organisation for Research and Treatment of Cancer 10981‐22023 AMAROS (After Mapping of the Axilla: Radiotherapy Or Surgery) trial had a 51 per cent rate of SLNB positivity, compared with 28 per cent of those with unifocal cancers^70^ ^71^.

Clinical surveillance informing cancer outcomes should optimally extend to at least 10 years^2^ ^3^. The Association of Breast Surgery guidelines^50^ recommend a target of 5‐year breast cancer LRR rates of 5 per cent or less from diagnosis. In future studies, the primary outcome measure for evaluating the impact of extent of surgery in MIBC should be 5‐year LRR, ideally with follow‐up to 10 years^72^, with disease‐specific and all‐cause mortality as important secondary outcomes^53^ ^72^. The results of meta‐analyses^72^ including a total of 10 800 patients from 17 randomized trials of RT *versus* no RT after BCS underscore this; RT reduced the 10‐year risk of any first recurrence (LRR and distant) by 16 per cent and breast cancer death by 4 per cent. Yerushalmi and colleagues^43^ reported a 5‐year LRR rate for MIBC of 2·5 per cent after BCS compared with 4·5 per cent after mastectomy. The discordant LRR rates in favour of BCS suggest biased case selection, with clinically more aggressive disease selected for mastectomy. A similar phenomenon is likely in six reported studies that compared LRR rates in women with MIBC treated with BCS *versus* mastectomy and were analysed here using a forest plot; this analysis showed no intrastudy heterogeneity and no apparent effect by type of surgery. Preliminary calculations suggest that, in future, comparable surgical groups should comprise at least 1000 patients each, based on predicted 5‐year LRR rates of 2·5 per cent^43^, highlighting that the currently available studies were underpowered.

Some of the reviewed studies^22^ ^27^ reported worse DFS and overall survival for MIBC than for single cancers, yet other studies^21^ ^43^ noted similar outcomes. MC cancers (but not MF cancers) were distinguished by significantly worse overall survival (*P* = 0·009) and DFS (*P* < 0·001) compared with unifocal cancers^22^. However, this was negated by a complete pathological response after neoadjuvant chemotherapy, independent of type of surgery^22^. Similarly, Wolters *et al*.^23^ reported a significant association between MIBC and relapse‐free survival in a study of 1862 MIBC compared with 7073 unifocal cancers (*P* = 0·007); however, this finding related to clinical non‐adherence to German guidelines. Weissenbacher and colleagues^19^ confirmed a significant association between MIBC and overall breast cancer recurrence (*P* = 0·001) in matched‐pair multivariable analyses of MIBC compared with unifocal cancers (288 in each group). These conflicting reports support a future review of current TNM staging for MIBC.

Molecular subtyping in breast cancers provides therapeutic and prognostic stratification^3^ ^17^. There is limited evidence on associations between MIBC and five molecular subtypes, compared with the subtype distribution in unifocal cancers^5^. A comprehensive IHC subtyping algorithm (six biomarkers) that can distinguish luminal B from luminal A cancers, and basal from triple‐negative disease, has potential clinical implications^15^ ^17^, ^18^. Luminal cancers had a lower risk of 5‐year LRR than HER2‐positive or triple‐negative unifocal disease after BCS in 12 500 patients^73^. Ataseven and co‐workers^22^ reported increased associations between oestrogen receptor‐positive and HER2‐positive genotypes in MIBC, compared with unifocal cancers (*P* < 0·001). Similarly, Moon *et al*.^74^ reported fewer triple‐negative MIBC than unifocal cancers. Lynch and colleagues^21^ showed no significant associations between MIBC (906 patients) and molecular subtypes. Given the growing appreciation of intertumoral heterogeneity in MIBC, molecular characterization of a single focus may underestimate the molecular landscape^12^. Standard phenotyping and genotyping of each cancer in MIBC should underpin future treatment recommendations.

The true biological and clinical significance of MIBC remains uncertain, with current expert consensus based on limited evidence. The studies reviewed here have historical limitations and were not adequately powered for conclusive treatment recommendations to be drawn based on LRR or survival after BCS compared with mastectomy. Meta‐analyses of existing historical prospective data sets or early randomized trials are likely to be beset by poor‐quality data on pathological focality. Despite the potential for use of TM to treat MIBC, the evidence base for readily adopting this treatment is poor. Valuable data on 5‐year effect size for LRR could derive from a current registry study (ACOSOG (American College of Surgeons Oncology Group) Z11102)^4^ ^75^. Based on this, an international collaboration guided by the IDEAL (Idea, Development, Exploration, Assessment, Long‐term Follow‐up) framework^51^ has suggested the need for a multicentre randomized trial (MIAMI trial)^75^. This trial should use a pragmatic classification of MF and MC cancers. In addition, the similarity or heterogeneity of genomic profiling for individual cancers is likely to supersede existing anatomical or surgical definitions of MIBC in the future.

## Supporting information


**Appendix S1** Search strategy
**Appendix S2** Data extraction pro‐forma
**Table S1** Summary of characteristics of papers reviewed and overall quality
**Table S2** Newcastle–Ottawa scale scoring
**Table S3** Clinical‐pathology characteristics and treatments
**Table S4** Clinical outcomes
