# Supplementary material for: Systematic review of the impact of breast‐conserving surgery on cancer outcomes of multiple ipsilateral breast cancers
Source: BJS Open. 2018 May 22;2(4):162–74. doi: 10.1002/bjs5.53 (PMC6069349; doi:10.1002/bjs5.53)
Supplement: Supplementary file 1 — Appendix S1 Search strategy Appendix S2 Data extraction pro‐forma Table S1 Summary of characteristics of papers reviewed and overall quality Table S2 Newcastle–Ottawa scale scoring Table S3 Clinical‐pathology characteristics and treatments Table S4 Clinical outcomes [file BJS5-2-162-s001.docx]

**BJS5_­53**

**Systematic review of the impact of breast-conserving surgery on cancer outcomes of multiple ipsilateral breast cancers**

Z. E. Winters, J. Horsnell, K. T. Elvers, A. J. Maxwell, L. J. Jones, A. M. Shaaban, P. Schmid, N. R. Williams, A. Beswick, R. Greenwood, J. C. Ingram, C. Saunders, J. S. Vaidya, L. Esserman, I. Jatoi and A. M. Brunt

**Appendix S1** Search strategy

**Search terms:** “breast cancer,” or “breast carcinoma,” or “Ductal carcinoma in situ,” or “DCIS,” and “multifocal,” or “multicentric,” or “multiple lesions,” or “multiple ipsilateral tumours” and “breast conservation,” or “wide local excision,” or “lumpectomy,” or “partial mastectomy”, or “quadrantectomy,” or “mammoplasty,” or “mastectomy,” or “breast reconstruction,” or “oncoplastic surgery”.

**‘Breast Cancer’**

| No. | Term |
| --- | --- |
| 1 | breast cancer/ |
| 2 | breast carcinoma/ |
| 3 | ductal carcinoma in situ/ |
| 4 | DCIS/ |
| 5 | 1 or 2 or 3 or 4 |
| 6 | Limit 5 to (English Language, humans) |

**‘Multifocal or Multicentric’**

| No. | Term |
| --- | --- |
| 1 | multicentric/ |
| 2 | multifocal/ |
| 3 | multiple lesions/ |
| 4 | multiple ipsilateral/ |
| 5 | 1 or 2 or 3 or 4 |
| 6 | Limit 5 to (English language, humans) |

**‘Breast surgery’**

| No. | Term |
| --- | --- |
| 1 | breast conservation/ |
| 2 | wide local excision/ |
| 3 | lumpectomy/ |
| 4 | partial mastectomy/ |
| 5 | quadrantectomy/ |
| 6 | mammoplasty/ |
| 7 | mastectomy/ |
| 8 | breast reconstruction/ |
| 9 | oncoplastic surgery |
| 10 | 1 or 2 or 3 or 4 or 5 or 6 or 7 or 8 or 9 |
| 11 | Limit 10 to (English language, humans) |

Combine searches 1 AND 2 AND 3

Limit studies to those published after May 1988

Remove duplicates

**Appendix S2** Data extraction pro-forma

**Review ID no:.**..............

**First Author:**………………………………. **Year:**.............................

**1. Type of study**

RCT □ Cohort study □ Case-series □

**2. Data collection**

Prospective □ Retrospective □ Not stated/unclear □

**3. Location of Study: (Country and city and institution)**:………………..

**Number of centres from which patients were recruited?**..........................

**4. Time period during which data was collected**?.................

**5.If RCT/Cohort study what was the comparison made?**

Breast conserving surgery vs. mastectomy (all patients with MIBC ) □

Breast conserving surgery in MIBC vs. breast conserving surgery in unifocal cancer □

**6. Sample Size (n=)**

MIBC total: ………… Multifocal: ………… Multicentric: ……….

Comparison group total: …………Multifocal: ……….. Multicentric: …………

**7. Were inclusion/exclusion criteria documented?**

Yes **□** No **□** Not clear **□**

**8. Was it clear how patients with MIBC were identified (ie pre-operative imaging, post-operative pathology)**

Yes **□** No **□** Not clear **□**

**9. Were the patients in the exposed cohort a true representation of the entire population?**

Yes **□** Partially **□** No **□** Not clear **□**

**10. In RCT/Cohort studies was the comparison group from the same population as the exposed group?**

Yes **□** No **□** Not clear **□**

**11. Was it possible to define the demographic/ pathological characteristics of the exposed and comparison group?**

Yes **□** No **□**

**12. If yes to 11, what were the characteristics recorded? What were the percentages in each group and were there statistically significant differences in each group?**

| **Descriptor** | **Exposed Group** | **Comparison Group** | **Significant Difference?** |
| --- | --- | --- | --- |
|  |  |  |  |
|  |  |  |  |
|  |  |  |  |
|  |  |  |  |
|  |  |  |  |
|  |  |  |  |
|  |  |  |  |

**13. Were statistical tests applied to account for any differences in 12?**

Yes **□** No **□** Not clear **□** Not applicable **□**

What tests were used? ………………………………………

**14. Adjuvant Treatments**

**a. Was the use of Radiotherapy documented?**

Yes **□** No **□** Not clear **□**

If yes what percentages in each group received radiotherapy and what regimes were used (including the use of Boost Radiotherapy)?

………………………………………………………………………………

**b. Was the use of Chemotherapy documented?**

Yes **□** No **□** Not clear **□**

If yes what percentages in each group received chemotherapy and what regimes were used?

……………………………………………………………………………….

**c. Was the use of Hormone Treatment documented?**

Yes **□** No **□** Not clear **□**

If yes what percentages in each group received adjuvant hormone treatment and what was used?

……………………………………………………………………………….

**15. Follow-up**

**a.** Duration of follow up …………………… Not stated **□**

**(**If follow-up different for different groups please state: ………………….)

**b.** What was the adequacy of follow-up

<10% of population lost to follow-up **□** > 10% of population lost to follow up **□** Not clear **□**

**16. Outcomes**

**a. What outcomes were recorded in the study?**…………………………………………………………………………….

**b. Was the assessment of outcome independent?**

Yes **□** No **□** Not clear **□**

**c. For each outcome measure record the result for each group (where applicable)**

| **Outcome** | **Exposed Group** | **Comparison Group** | **Significant Difference?** |
| --- | --- | --- | --- |
|  |  |  |  |
|  |  |  |  |
|  |  |  |  |
|  |  |  |  |
|  |  |  |  |
|  |  |  |  |
|  |  |  |  |

**Table S1** Summary of characteristics of papers reviewed and overall quality

**A) Case-series**

| **Paper** | **No. Recruiting Centres** | **Data collection** | **Study design** | **No. MFBC treated by BCS** | **No. MF treated by BCS** | **No. MC treated by BCS** | **No. comparator group*** | **Method detection MIBCs**  **(no.)** | **Inclusion Criteria** | **Clinical Outcomes**** | **Follow-up months (median unless stated: (IQR))** | **Newcastle-Ottawa Score^** |
| --- | --- | --- | --- | --- | --- | --- | --- | --- | --- | --- | --- | --- |
| Hartsell^44^ | Single centre | 1977-1989 | Retrospective | 27 | n/a | n/a | n/a | Clinical (2) Imaging (2) Path (23) | Yes | LR, DFS, OS | 53  (29-84) | n/a |
| Cho^45^ | Two centres | 1989-1997 | Retrospective | 15 | n/a | n/a | n/a | Clinical (2) Imaging (7) Path (6) | Yes | LRF, DM | 77  (40-130) | n/a |
| Gentilini^28^ | Single centre | 1997-2002 | Retrospective | 476 | 421 | 55 | n/a | n/d | Yes | CLE, CM | 73  (11-118) | n/a |
| Chie^46^ | Single centre | 1996-2002 | Retrospective | 13 | 10 | 3 | n/a | Clinical (6) Imaging (3) Path (3) | Yes | LR, Cosm. | 70  (46-119) | n/a |
| Bauman^47^ | Single centre | 1998-2008 | Retrospective | 22 | n/a | n/a | n/a | Clinical (3) Imaging (19) | Yes | LRR, DD | 42  (mean)^^ | n/a |
| Eryilmaz^48^ | Single centre | 1999-2003 | Retrospective | 59 | 55 | 4 | n/a | Path (59) | Yes | DFS, OS | 20  (2-97) | n/a |
| Kapoor^49^ | Single centre | 1998-2008 | Retrospective | 7 | nil | 7 | n/a | Imaging (7) | Yes | LRR, DD OS | 26  (18-85) | n/a |

**B) Retrospective cohort studies comparing BCS versus mastectomy for MIBC**

| Nos^32^ | Single centre | 1983-1989 | Retrospective | 56 | 56 | MC excluded | 132 | Clinical (14) Intra-op. (28)  Path. (14) | Yes | LR, DM, OS | 101  (86-129) | 5 |
| --- | --- | --- | --- | --- | --- | --- | --- | --- | --- | --- | --- | --- |
| Kaplan^33^ | Two centres | 1989-2002 | Retrospective: Prospective DB | 36 | n/a | n/a | 19 | Diagnosed  Pre-op. (36) | Yes | LR, DM | 45  (1-143) | 5 |
| Lim*** ^34^ | Single centre | 1990-2003 | Retrospective | 147^e^ | 147^e^ | nil | 331 | n/d | Yes | LRR, DFS OS | 59 (mean)  (1-177) | 6 |
| Wolters^23^ | 17 centres | 1992-2008 | Retrospective | 683^i^ | 623^i^ | 60^i^ | 329 (MF by mastectomy)  217 (MC by mastectomy)^i^ | n/d | Yes | RFS | 55 (MF)  54 (UF)  50 (MC)^^ | 5 |
| Kadioglu^35^ | Single centre | 2002-2011 | Retrospective | 119 | 119 | nil | 103 | Clinical (53) Imaging (24) Path. (40) | Yes | LR, OS | 55  (10-102) | 6 |
| Neri*** ^20^ | Single centre | 1991-2005 | Retrospective: Prospective DB | 36 | 36 | nil | 155 | n/d | Yes | LR, RR, DR | 88  (11-248) | 6 |

**C) Retrospective cohort studies comparing BCS for MIBC and BCS for UFC**

| **Paper** | **Number recruiting centers** | **Data- collection** | **Study design** | **No. MFBC treated by BCS** | **No. MF treated by BCS** | **No. MC treated by BCS** | **No. comparator group*** | **Method detection MFBCs**  **(no.)** | **Inclusion Criteria** | **Clinical Outcomes**** | **Follow-up months (median unless stated: (IQR))** | **Newcastle-Ottawa Score^** |
| --- | --- | --- | --- | --- | --- | --- | --- | --- | --- | --- | --- | --- |
| Leopold^36^ | Single centre | 1968-1981 | Retrospective | 10 | n/a | n/a | 707 | Clinical (7)  Imaging (1), Path. (2) | Yes | LRR, DM Cosm. | 64  (17 -115) | 5 |
| Kurtz^37^ | Single centre | 1975-1983 | Retrospective | 61 | n/a | n/a | 525 | Clinical (20)  Imaging (2), Path. (39) | Yes | LR | 71^^ | 5 |
| Wilson^38^ | Two centres | Before 1988 | Retrospective | 13 | n/a | n/a | 990 | Clinical (2)  Imaging (3)  Intra-op. (4)  Path. (4) | Yes | LR, OS | 71^^ | 5 |
| Okumura^39^ | Two centres | 1993-1999 | Retrospective | 34 | n/a | n/a | 594 | Pre-operative (26) Intra-op. (8) | Yes | LR, Cosm. | 58  (38-105) | 5 |
| Oh^40^ | Single centre | 1977-2003 | Retrospective | 20^b^ | n/a | n/a | 260 | Clinical (20) | Yes | LRR, OS | 66 (mean)^^ | 5 |
| Rakovitch^41^ | Single centre | 1982-2000 | Retrospective | 153^c^ | 153^c^ | nil | 137^c^ | n/d | Yes | LR | 59  (1-235) | 6 |
| Cabioglu^42^ | Single centre | 1990-2002 | Retrospective | 30^d^ | n/a | n/a | 498^d^ | n/d | Yes | LR, DFS | 48  (13-145) | 4 |
| Lim*** ^34^ | Single centre | 1990-2003 | Retrospective | 147^e^ | 147^e^ | nil | 930 | n/d | Yes | LRR, DFS OS | 59 (mean;1-177) | 5 |
| Chung^27^ | Single centre | 1991-2009 | Retrospective: Prospective DB | 164 | 164 | nil | 999 | Imaging (35)  Path. (124)^a^ | Yes | LRR, DFS OS | 112  (1-230) | 6 |
| Yerushalmi^43^ | Regional Cancer Agency | 1989-2005 | Retrospective: Prospective DB | 300^f^ | n/a | n/a | 11683^f^ | n/d | Yes | LRR | 93^^ | 7 |
| Ataseven^22^ | Trial participants | 2002-2010 | Retrospective review of trial data | 617^g^ | 454^g^ | 163^g^ | 3217^g^ | n/d | Yes | LRFS, DFS OS | 36  (0-110) | 4 |
| Lynch^21^ | Single centre | 1997-2010 | Retrospective: Prospective DB | 256^h^ | 256^h^ | nil ^h^ | 1757 | Path. (256) | Yes | LRR | 52  (1-152) | 5 |
| Neri*** ^20^ | Single centre | 1991-2005 | Retrospective: Prospective database | 36 | 36 | nil | 491 | n/d | Yes | BCSS, LR RR, DR | 88  (11-248) | 6 |

MIBC = Multiple ipsilateral breast cancer, MF= Multifocal, MC = Multicentric, BCS = Breast conserving surgery, UFC = Unifocal cancer, LR=Local Recurrence, DFS = Disease Free Survival, OS = Overall Survival, LRF = Local regional failure, DM = Distant Metastases, CLE = Cumulative local events, CM = Cumulative Mortality, Cosm. = Cosmetic appearance, No. = Numbers of patients, Op. = Operative, Path. = Pathology, IQR = inter-quartile range, DB. = Database, LRR = Loco-regional recurrence, DD = Distant Disease, RFS = Recurrence Free Survival, LRFS = Local Relapse Free Survival, BCSS = Breast Cancer specific survival, LR = local recurrence, RR = Regional recurrence, DR = Distant recurrence. N/a = not applicable. N/d = not able to determine this from the data given in the paper. ^^ = No details given regarding SD. (Standard deviation) or range.

*Either MIBC treated by mastectomy or unifocal cancer treated by BCS, ** Table 3 describes clinical-pathological characteristics, ***Lim^34^ and Neri^20^ papers appear twice, containing two sets of comparative data: BCS versus Mastectomy for MFBC and BCS for MFBC versus UFC. ^ Scoring Quality by the Newcastle-Ottawa Scale, where quality is classified as: High 7-9 stars, Moderate 4-6 stars and poor less than 4 stars.

Superscripts: A = imaging data missing for 5 patients, B = 97 patients with MFBC with 20 treated by BCS. C = Exclusive treatment by BCS and radiotherapy, D = 30 Patients out of 147 with MFBC treated by BCS and compared to 498 UFC treated by BCS. E = 478 patients with MF disease where 147 underwent BCS. F = 1187 patients with MIBC, where 300 underwent BCS.

G = 1401 patients with MIBC with 617 treated by BCS. H = 906 patients with MF or MC, with 256 MF only treated by BCS. I = 8935 patients and 1862 with MIBC, however only a small data subset (MIBC treated by BCS and radiotherapy) was extractable and relevant.

**Table S2** Newcastle–Ottawa scale scoring

| **Newcastle-Ottawa Scale** | **Selection  (Max 4)** | | | | **Comparability  (Max 2)** | **Outcomes (Max 3)** | | | **Overall Score** |
| --- | --- | --- | --- | --- | --- | --- | --- | --- | --- |
| Paper | Representation of exposed cohort -True Representation * -Partial Representation * -Selected Group -Not clear | Selection of the non-exposed cohort -from same community * -different source -Not clear | Ascertainment of exposure -secure record -no report | Demonstration that outcome of interest not present at start | Study controls for Disease-stage/ Biological features | Assessment of outcome -Independent  - record linkage | Was follow-up long enough (at least 5 years) | Adequacy of  Follow-up -complete*  <10% lost >10% lost Not clear | Max Score  = 9 |
| Yerushalmi^43^ | * | * | * | * | ** |  | * |  | 7 |
| Rakovitch^41^ | * | * | * | * |  |  | * |  | 5 |
| Lim^34^  (BCS MFBC  vs.Mx MFBC) | * | * | * | * |  |  |  |  | 4 |
| Chung^27^ | * | * | * | * | * | * | * |  | 7 |
| Kadioglu^35^ | * | * |  | * | ** |  |  |  | 5 |
| Leopold^36^ |  | * | * | * |  |  | * |  | 4 |
| Kurtz^37^ | * | * | * | * |  | * | * |  | 6 |
| Wilson^38^ | * | * | * | * |  |  | * |  | 5 |
| Nos^32^ |  | * | * | * |  |  | * |  | 4 |
| Kaplan^33^ | * | * | * | * |  |  |  |  | 4 |
| Okumura^39^ | * | * | * | * |  |  |  |  | 4 |
| Oh^40^ | * | * | * | * |  |  | * |  | 5 |
| Lynch^21^ | * | * | * | * |  |  |  |  | 4 |
| Wolters^23^ | * | * | * | * | * |  |  |  | 5 |
| Cabioglu^42^ | * | * | * | * |  |  |  |  | 4 |
| Ataseven^22^ | * | * | * | * |  | * |  |  | 5 |
| Neri^20^ | * | * | * | * | ** |  | * |  | 7 |

Quality scoring assessments using the Newcastle-Ottawa Scale, where quality is classified into three levels: High 7-9 stars; Moderate 4-6 stars and poor less than 4 stars. Abbreviations: Max. = Maximum

**Table S3** Clinical-pathology characteristics and treatments

**A) Retrospective cohort studies of MIBC treated by BCS versus mastectomy**

| **Paper** | **Pathology MIBC treated by BCS** | **Pathology comparison group*** | **Clinical-pathology MIBC treated by BCS & comparison group*** | **Significant intergroup differences** | **Statistics**  **Intergroup differences**** | **RT treatment***** | **Chemotherapy treatment***** | **Endocrine treatment***** |
| --- | --- | --- | --- | --- | --- | --- | --- | --- |
| Nos^32^ | IDC - 44/56 (79%) ILC - 7 /56(13%) Others - 5/56 (9%) | IDC - 110/132(83%) ILC - 11/132 (8%) Others - 11/132 (8%) | Age, menopause,  T, N, histology, ER | Age (p<0.0001),  T (p<0.001) | No | Yes | Yes | Yes |
| Kaplan^33^ | IDC - 26/36 (72%) ILC - 7/36 (19%) DCIS - 3/36 (8%) | IDC - 13/19 (68%) ILC - 4/19 (21%) DCIS - 2/19 (11%) | Age, T, grade, N, ER, race, histology | Nil | n/a | Yes | Yes | Yes |
| Lim^34^ | IDC 142/147(97%) ILC - 5/147 (3%) | IDC - 317/331(96%) ILC - 14/331 (4%) | Age, menopause  T, grade, ER,  HER2, EIC,  no. cancers,  histology | HER-2 (p=0.007) | Yes TNM | Yes^ | No | Yes |
| Wolters^23^ | n/d | n/d | n/d | n/a | Yes  T (for MC only) | Yes | No | No |
| Kadioglu^35^ | IDC - 85/119(71%) ILC - 14/119(12%) Mixed-12/119(10%) Other –8/119 (7%) | IDC - 71/103 (69%) ILC - 8/103 (8%) Mixed – 16/103 (16%) Other – 8/103 (7%) | Menopause,  TN, TNM, ER, PR  HER-2, no. cancers,  LVI, family-history, histology | no. cancers  (p=0.01), N (p=0.002),  TNM (p=0.01), HER-2 (p=0.03) | Yes TNM | Yes | Yes | Yes |
| Neri^20^ | n/d | n/d | n/d | n/d | n/a | Yes | Yes | Yes |

**B) Retrospective cohort studies comparing BCS for MIBC and BCS for UFC**

| **Paper** | **Pathology MIBC treated by BCS** | **Pathology comparison group*** | **Clinical-pathology MIBC treated by BCS & comparison group*** | **Significant intergroup differences** | **Statistics**  **Intergroup differences**** | **RT treatment***** | **Chemotherapy treatment***** | **Endocrine treatment***** |
| --- | --- | --- | --- | --- | --- | --- | --- | --- |
| Leopold^36^ | IDC - 8/10 (80%) Mixed - 2/10 (20%) | IDC - 509/707 (72%) Other - 198/707 (18%) | Histology, EIC | Nil | n/a | Yes | No | No |
| Kurtz^37^ | IDC - 54/61 (89%)  ILC - 3/61 (5%)  DCIS - 3/61(5%) Other - 1/61 (2%) | IDC - 442/525 (84%)  Other - 83/525 (16%) | Age, histology, T, EIC, ER,  N, margins | Margins (p=0.01) | No | No | No | No |
| Wilson^38^ | IDC - 11/13 (85%) Lobular - 2/13 (15%) | n/d | n/d | n/d | n/a | Yes | Yes | No |
| Okumura^39^ | IDC - 29/34 (85%) ILC - 3/34 (9%) Other - 2/34 (6%) | IDC - 511/594 (86%) ILC - 27/594 (5%) DCIS - 22/594 (4%)  Others 34/594 (6%) | Age, T, N, ER, EIC, margins  RT boost field-size, histology | Margins (p<0.01) | No | Yes | Yes | Yes |
| Oh^40^ | n/d | n/d | Age, T, grade, N, ER, TNM | Nil | n/a | Yes | All NAC | No |
| Rakovitch^41^ | DCIS - 153/153 (100%) | DCIS - 137/137 (100%) | n/d | n/d | n/a | Yes | n/a | Yes |
| Cabioglu^42^ | n/d | n/d | n/d | n/d | n/a | No | Yes^^ | Yes |
| Lim^34^ | n/d | n/d | n/d | n/d | n/a | Yes ^ | No | Yes |
| Chung^27^ | IDC - 96/164 (59%) ILC - 40/164 (24%)  Mixed - 27/164 (17%) Other 1/164% | IDC - 816/999 (82%) ILC - 75/999 (8%)  Mixed - 100/999 (10%) Other - 8/999 (1%) | Age, histology,  T, grade, N, ER, TNM | Histology (p<0.0001),  T (p=0.001),  TNM (p<0.0001) | Yes T, N | Yes | Yes^^ | Yes |
| Yerushalmi^43^ | IDC 88%^^^^^ ILC 12%  Other 1% | IDC 92% ^^^^^ ILC 6%  Other 1% | Age, T, grade, N, ER, LVI  EIC, margins, histology | T (p=0.005),  N (p=0.01), EIC (p<0.001), margins (p=0.003) | Yes Case-match: TNM, LVI, ER, DCIS, histology, margins | Yes | Yes | Yes |
| Ataseven^22^ | n/d | n/d | n/d | n/d | n/a | No | All NAC | No |
| Lynch^21^ | n/d | n/d | n/d | n/d | n/a | Yes | No | Yes |
| Neri^20^ | n/d | n/d | n/d | n/d | n/a | yes | Yes | Yes |

EIC = Extensive ductal cancer in situ (DCIS), LVI = Lymphovascular invasion, ER = Estrogen Receptor, PR = Progesterone Receptor, HER-2 = Human Epidermal Growth Factor Receptor, NAC = Neoadjuvant chemotherapy,

IDC = Invasive Ductal Cancer, ILC = Invasive Lobular cancer, DCIS = Ductal Carcinoma in Situ, LCIS = Lobular carcinoma in situ, RT = Radiotherapy, T = Tumour size (T), N = Lymph node status, Stage = TNM,

MIBC = Multiple ipsilateral breast cancers, MF = Multifocal, MC = Multicentric, UFC = Unifocal, BCS = Breast conserving surgery, UFC = unifocal cancer, Grade = Histological grade, Margins = resection margins,

No. = numbers of cancers. N/a = not applicable, N/d = not able to determine (data precluded subgroup determination or was not presented).

*MIBC treated by mastectomy versus UFC treated by BCS. ** Yes for stratification of factors listed: Worse factors for Mastectomy (A) and for MIBC (B). ***Yes for details of the percentages of patients receiving the treatment.

^ Although numbers within the separate UFC and MFBC groups are unclear. ^^ Incomplete data presented and acknowledged in the paper. ^^^^^ Numbers of patients not given.

**Table S4** Clinical outcomes

**A) Case-series**

| **Paper** | **Follow-up (months)** | **Local recurrence*   (LR, LRR , LRF, CLE)** | **Disease-free survival* (RFS, DFS)** | **Distant metastases* (DM /DD)** | **Overall survival*  (OS, BCSD, OM, BCSS)** |
| --- | --- | --- | --- | --- | --- |
| Hartsell^44^ | 53 | 3.7% (1/27) | n/r | 11% (3/27) | 4-year actuarial 89% |
| Cho^45^ | 77 | 0% | n/r | 7% | n/r |
| Gentilini^28^ | 73 | 6-year actuarial 5.1% | n/r | n/r | 5-year actuarial 94% |
| Chie^46^ | 70 | 0% | n/r | n/r | n/r |
| Bauman^47^ | 42 | 4.50% (1/22) | n/r | 4.50% (1/22) | n/r |
| Eryilmaz^48^ | 20 | n/r | 5-year actuarial 92.3% | n/r | 5-year actuarial 95% |
| Kapoor^49^ | 26 | 0% | n/r | 0% | 100% |

**B) Retrospective cohort studies comparing BCS to mastectomy in MIBC**

| **Paper** | **Follow-up (months)** | **Local recurrence*   (LR, LRR, LRF, CLE)** | **Disease-free survival* (RFS, DFS)** | **Distant metastases* (DM /DD)** | **Overall survival* (OS, BCSD, OM, BCSS)** |
| --- | --- | --- | --- | --- | --- |
| Nos^32^ | 101 | 5-year actuarial 11.2% vs. 11.5 %  10-year actuarial 22.8% vs.14.2%  p value n/r | n/r | 5-year actuarial 18.1% vs. 18.3% 10-year actuarial 28% vs. 35%  p value n/r | 5-year actuarial 94.4% vs. 89.8%  10-year actuarial 73.3% vs. 65.1%  p value n/r |
| Kaplan^33^ | 60 | 3% (1/36) vs. 0% (0/19)  p=0.54 | n/r | 3% (1/36) vs. 5% (1/19)  p=0.20 | 100% vs. 100% |
| Lim^34^ | 59.3 | 2.0% (3/147) vs. 0.9% (3/331)  p=0.378 | 5-year actuarial 89.08% vs. 91.88%  p=0.451 | n/r | 5-year actuarial 93.38% vs.94.53%  p=0.208 |
| Wolters^23^ | 55 | n/r | MF = HR 1.25 (95% CI 0.83 - 1.88)  MC = HR 1.23 (95% CI 0.51 - 3.0)  p=0.284 | n/r | n/r |
| Kadioglu^35^ | 55 | 5% (6/119) vs. 5.8% (6/103)  p=0.06 | n/r | n/r | 92% vs. 72%  (p<0.001)  no raw data/curves |
| Neri^20^ | 88 | LR: 8.3% (3/36) vs.7.7 (12/155)  RR: 5.6% (2/36) vs. 7.1% (11/155) | n/r | 19.4% (7/36) vs. 27.1% (42/155)  p value n/r | n/r |

**C) Retrospective cohort studies comparing BCS for MIBC to BCS for UFC**

| **Paper** | **Follow-up (months)** | **Local recurrence***  **(LR, LRR, LRFS, CLE)** | **Disease-free survival* (RFS, DFS)** | **Distant metastases***  **(DM, DD)** | **Overall survival *  (OS, BCSD, OM, BCSS)** |
| --- | --- | --- | --- | --- | --- |
| Leopold^36^ | 64 (MFBC)  75 (UFC) | 40% (4/10) vs. 11% (77/707) p=0.019 | n/r | 40% (4/10) vs. 22% (152/694)  p=0.223 | n/r |
| Kurtz^37^ | 71 | 25% (15/61) vs. 11% (56/525) (p<0.005) | n/r | n/r | n/r |
| Wilson^38^ | 71 | 6-year actuarial 25% + 0.16 vs.12% + 0.12  p value n/r | n/r | n/r | 6-year actuarial 81% + 0.12 vs.85% + 0.02  p value n/r |
| Okumura^39^ | 58 | 2.9% (1/34) vs. 2.4% (15/564)  p value n/r | n/r | n/r | n/r |
| Oh^40^ | 66 | 5-year actuarial 6% vs. 11%  p=0.78 | n/r | n/r | 5-year actuarial 86% vs. 83%  p value n/r |
| Rakovitch^41^¶ | 59 | 5-year actuarial 8% vs. 6% p=0.41 10-year actuarial 19% vs. 16% p=0.40 | n/r | n/r | n/r |
| Cabioglu^42^ | 48 (MFBC)   56 (UFC) | 6.7% (2/30) vs. 5.4% (27/498)  p=0.68 | n/r | n/r | n/r |
| Lim^34^ | 59.3 | 2% (3/147) vs. 1.3% (12/930)  p=0.445 | 5-year actuarial 89.08% vs. 90.69%  p=0.451 | n/r | 5-year actuarial 93.38% vs. 93.60%  p=0.208 |
| Chung^27^ | 112 | 6.1% (10/164) vs. 0.6% (6/999)  HR 23.87 (CI5.81,98.11)  p<0.0001 | 89.3% (14/164) vs. 97.7% (17/999)  HR 5.86 (CI 2.57,13.3)  p<0.0001 | n/r | 85.8% (12/164) vs. 98.4% (11/999)  HR 10.57 (CI 4.2, 26.59)  p<0.0001 |
| Yerushalmi^43^† | 93 | LR = 5.6% vs. 4.3%  HR 1.09 (CI 0.55, 2.16)  p=0.78  LRR = 6.8% vs. 4.8%  HR 1.09 (CI 0.55, 2.16)  p=0.60 | n/r | n/r | n/r |
| Ataseven^22^ | 36 | MF = 4.2% (19/454)  MC = 8.0% (13/163) vs. UFC = 5.7% (182/3217)  p=0.314 | MF = 86.8% (60/454)  MC = 81.6% (30/163)  vs.  UFC = 85.6% (463/3217)  p=0.546 | n/r | n/r |
| Lynch^21^ | 52 | 1.95% %/ (5/256) vs. 1.02% (18/1757)  p=0.67 | n/r | n/r | n/r |
| Neri^20^ | 88 (11-248) | LR = 8.3% (n=3/36) vs. 5.9% (n=12/155)  p=0.32  LRR = 5.6% (2/36) vs. 1.6% (8/491)  p=0.05 | n/r | 19.4% (7/36) vs.11.2% (55/491)  p=0.03 | BCSS >BCS for MIBC  HR 3.88 (CI 1.06-14.12)  p=0.02 |

MIBC = Multiple ipsilateral breast cancers, MF = Multifocal, MC = Multicentric, UFC = Unifocal cancer, BCS = Breast conserving surgery, LR = Local Recurrence, DFS = Disease free survival, OS = Overall survival, LRF = Local regional failure, DM = Distant metastases, CLE = Cumulative local events, CM = Cumulative mortality, Cosm. = Cosmetic, LRR = Loco-regional recurrence, RR = Regional recurrence, DD = Distant disease, RFS = Recurrence-free survival, LRFS = Local relapse free survival, BCSD = Breast cancer - specific death, BCSS = Breast cancer - specific survival, OM = Overall mortality, HR = Hazard ratio, NS = Not statistically significant, N/a = not applicable, N/r = not reported as an outcome, Yrs. = Years, Vs. = Versus. *Percentage at the maximum follow-up, unless otherwise stated (comparison group figures are presented second). ¶ Rakovitch^41^ primarily aimed to evaluate MF and MC ductal carcinoma in situ, but included invasive cancers (Table S4, supporting information). †Yerushalmi^43^ includes a matched analysis.
